# Comparative physiological and genomic characterization of a novel *Nitrobacter vulgaris* strain from a nitrate-contaminated subsurface

**DOI:** 10.1128/aem.00130-26

**Published:** 2026-03-24

**Authors:** Zachary Flinkstrom, Kristopher A. Hunt, Britt Abrahamson, Pierce Harvell, Xiangpeng Li, James Wilson, Zachary S. Cooper, Jacob J. Valenzuela, Nitin S. Baliga, David A. Stahl, Wei Qin, Mari-Karoliina H. Winkler

**Affiliations:** 1 Department of Civil and Environmental Engineering, University of Washington 7284, Seattle, Washington, USA; 2 Institute for Systems Biology 7268, Seattle, Washington, USA; 3 School of Biological Sciences, Institute for Environmental Genomics, University of Oklahoma 6187, Norman, Oklahoma, USA; 4 Department of Microbiology, School of Molecular and Cellular Biology, Carl R. Woese Institute for Genomic Biology, University of Illinois Urbana-Champaign 14589, Urbana, Illinois, USA; University of Minnesota Twin Cities, St. Paul, Minnesota, USA

**Keywords:** nitrite-oxidizing bacteria, nitrification, *Nitrobacter*, nitrate toxicity, nitrite toxicity, nitrous oxide, comparative genomics, kinetics

## Abstract

Nitrite-oxidizing bacteria (NOB) represent a crucial node in the global nitrogen cycle. By catalyzing the second step of nitrification—the oxidation of nitrite to nitrate to generate energy for growth—NOB activity controls the fate of nitrite (NO_2_^-^) in aerobic environments. Despite thriving in diverse environments, including soils, freshwater, marine ecosystems, subsurface habitats, and water treatment systems, organisms capable of nitrite oxidation are confined to *Nitrobacter, Nitrospira, Nitrospina, Nitrotoga,* and a few other specific lineages. The genus *Nitrobacter*, recognized for its facultative heterotrophic metabolism, is often associated with high-nitrogen environments. Here, we report the physiological characterization of a novel strain, *Nitrobacter vulgaris* strain MLSD-S22, isolated from a nitrate- and heavy-metal-contaminated subsurface. Growth inhibition experiments revealed that strain MLSD-S22 and the *N. vulgaris* type strain Z exhibited similar sensitivities to nitrite and nitrate, with nitrite being the most inhibitory. Microrespirometry demonstrated that the two *N. vulgaris* strains and *Nitrobacter winogradskyi* Nb-255 possessed higher affinities for nitrite and oxygen than previously reported for *Nitrobacter*, suggesting potential to compete in low-substrate environments. Long-read DNA sequencing provided a complete genome for strain MLSD-S22, revealing two plasmids and an intact nitrous oxide (N_2_O) reduction operon—an unexpected feature for *Nitrobacter*. While N_2_O reduction activity was not observed under the tested conditions, this discovery raises questions about the contribution of *Nitrobacter* NOB to the N_2_O sink. These findings broaden the physiological and genomic diversity of *Nitrobacter*, offering new insights into their adaptation strategies and providing a framework for future evaluation of their potential roles in nitrogen loss.

**IMPORTANCE:**

*Nitrobacter* species play a key role in nitrogen cycling; however, their physiological adaptations and genomic diversity remain largely underexplored. This study characterizes *Nitrobacter vulgaris* strain MLSD-S22, isolated from a nitrate- and heavy-metal-contaminated subsurface, revealing unexpected genomic and metabolic traits. Higher nitrite and oxygen affinities compared to previous reports indicate that some *Nitrobacter* are more competitive in low-substrate environments than previously believed. The presence of a complete nitrous oxide (N_2_O) reduction operon highlights the genomic potential related to N_2_O reduction within this genus; however, we did not observe N_2_O reduction under the conditions tested. By expanding *Nitrobacter* ecophysiology, this work provides a foundation for future research on the metabolic flexibility of *Nitrobacter* and their contributions to nitrogen transformations in diverse environments.

## INTRODUCTION

Nitrite-oxidizing bacteria (NOB) represent a diverse group of chemolithoautotrophs that conserve energy by catalyzing the second step of nitrification—the oxidation of nitrite (NO_2_^−^) to nitrate (NO_3_^−^). These organisms play an important role in the global nitrogen cycle by controlling the availability of NO_2_^−^, a key intermediate in nitrification, denitrification, dissimilatory reduction of nitrate to ammonium (DNRA), and anaerobic ammonium oxidation (anammox) (1). Environmental factors, including temperature and dissolved oxygen (O_2_), can decouple the activities of ammonia-oxidizing microorganisms (AOM) and NOB, leading to the accumulation of NO_2_^−^ (2–4). While NOB inhibition may be favorable in the context of partial nitritation (i.e., halting nitrification at the conversion of ammonia to nitrite) or nitrite shunt systems in wastewater treatment (4), NO_2_^−^ accumulation has implications for environmental toxicity (5) and the emission of nitrous oxide (N_2_O) (6, 7)—a potent greenhouse gas with approximately 265 times the global warming potential of carbon dioxide (CO_2_) over a 100-year time period (8). Given the significance of NOB, it is important to understand the environmental factors controlling their activity and contribution to nitrogen transformation and nitrogen loss.

Currently, NOB are classified into seven genera across four bacterial phyla: *Pseudomonadota*, *Nitrospirota*, *Nitrospinota*, and *Chloroflexota* (1). The genus *Nitrobacter*, within the class *Alphaproteobacteria* and phylum *Pseudomonadota*, is widely distributed across diverse environments such as soil, wastewater, river water, and sand filters and includes multiple representative isolates (9). The capacity of *Nitrobacter* NOB to supplement their catabolic and anabolic requirements with organic carbon (i.e., mixotrophy or heterotrophy) is well documented (1, 10–12). Although this trait is common in *Nitrobacter*, mixotrophic or heterotrophic growth has also been reported in other NOB lineages, including members of *Nitrospira* and *Nitrococcus* (13–16). Given the low energy yield of nitrite oxidation (ΔG°′ = −74 kJ/mol NO₂^−^), it is unsurprising that NOB exhibit metabolic versatility to persist in fluctuating environmental conditions.

Differences in nitrite oxidation kinetics across NOB clades contribute in part to their niche differentiation (17). *Nitrospira* NOB generally have higher affinities for NO₂^−^ compared to *Nitrobacter* and are considered oligotrophs, thriving under NO₂⁻-limiting conditions (1, 17). In contrast, *Nitrobacter* are generally thought to be copiotrophs, preferring environments with high NO₂^−^ concentrations (18). The two genera also exhibit pronounced differences in the biochemistry and localization of the nitrite oxidoreductase (NXR) complex. *Nitrospira* has a periplasmic-facing NXR, while the NXR active site in *Nitrobacter* is cytoplasmic-facing (1). Consequently, *Nitrobacter* must transport NO₂^−^ into the cytoplasm prior to NO₂^−^ oxidation. Furthermore, the protons released from cytoplasmic NO₂⁻ oxidation do not contribute directly to proton motive force generation, thereby reducing the ATP yield of *Nitrobacter* compared to *Nitrospira*. Carbon fixation pathways lead to further differences in the cellular yield of these genera. *Nitrobacter* fixes carbon using the Calvin–Benson–Bassham (CBB) cycle, while *Nitrospira* utilizes the reductive tricarboxylic acid cycle (rTCA), which has a higher carbon fixation yield per NO_2_^−^ oxidized (19). Taken together, these physiological and biochemical differences highlight distinct ecological strategies among NOB.

The Oak Ridge Reservation (ORR) in Tennessee contains a contaminated groundwater site that presents an example of an environment where NOB may require specific adaptive traits to persist and grow. This site is heavily contaminated with nitric acid and a mixture of heavy metals (aluminum, manganese, nickel, and uranium). In this environment, the concentration of NO_3_^−^ can be exceptionally high (up to 190 mM), and the pH can be as low as 3, although the area used in this study was closer to 6.5 (20, 21). Elucidating how NOB and other microorganisms adapt to these challenging conditions, in terms of their genomes and physiological traits, is crucial for revealing how the nitrogen cycle is affected in harsh geochemical settings.

To investigate potential adaptations to environmental stressors, we enriched and isolated a *Nitrobacter* strain from the ORR contaminated site and compared the physiology and genome to strains from non-contaminated sources. We assessed its growth response to elevated concentrations of NO₃^−^ and NO₂^−^ to determine whether it was adapted to resist these stressors in the environment. Kinetic analyses of NO₂^−^ and O₂ consumption revealed relatively high substrate affinities for *Nitrobacter*. Additionally, genomic sequencing uncovered two plasmids and an unexpected putative operon for N₂O reduction, although the activity was not demonstrated under the tested conditions. This study provides a comprehensive physiological and genomic characterization of a novel *Nitrobacter* isolate, expanding our understanding of genomic and metabolic versatility within the genus.

## MATERIALS AND METHODS

### Enrichment and isolation

NOB were enriched from a nitrifying bioreactor fed with 1 mM ammonium chloride and operated at a dilution rate of 0.02 h^−1^ for 1 year with a headspace of 5% O_2_:15% CO_2_: 80% N_2_ at 20°C and pH 6.9 ± 0.2. The bulk liquid of the chemostat-like reactor was used to fluidize a sediment bed of 60 g of 140–200 mesh quartz sand. The reactor was seeded with groundwater from well MLSD at the Oak Ridge Reservation site after three batch transfers. After the first 4 months of establishing process stability, NO_2_^-^ levels in the reactor remained consistently low (< 7 µM), similar to the field site, suggesting that the enriched NOB had high affinity for NO_2_^−^ and high turnover rates. To select for oligotrophic NOB species, only 0.1 mM NO_2_^−^ was added during the initial batch enrichment process, a concentration 10- to 100-fold lower than those employed in most previous NOB enrichment studies (9, 22–24).

A liquid sample from the bioreactor was inoculated for further enrichment in a minimal mineral medium prepared by adding (per liter) 1.0 g NaCl, 0.4 g MgCl_2_•6H_2_O, 0.1 g CaCl_2_•2H_2_O, and 0.5 g KCl, and autoclaving; then, the following were aseptically supplemented by filter-sterilization: 3 mL NaHCO_3_ (1 M), 5 mL KH_2_PO_4_ (0.4 g/L), 1 mL FeNaEDTA (7.5 mM), 1 mL trace elements solution, 1 mL vitamin solution, and 0.1 mL NaNO_2_ (1 M). The trace elements solution contained (per liter) 30 mg H_3_BO_3_, 100 mg MnCl_2_•4H_2_O, 190 mg CoCl_2_•6H_2_O, 24 mg NiCl_2_•6H_2_O, 2 mg CuCl_2_•2H_2_O, 144 mg ZnSO_4_•7H_2_O, 36 mg Na_2_MoO_4_•2H_2_O, and 8.4 mL concentrated HCl. The vitamin solution contained (per liter) 20 mg biotin, 20 mg folic acid, 100 mg pyridoxine HCl, 50 mg thiamine HCl, 50 mg riboflavin, 50 mg nicotinic acid, 50 mg DL-pantothenic acid, 50 mg p-aminobenzoic acid, 2 g choline chloride, and 10 mg vitamin B_12_. To enrich NOB with distinct pH adaptations, the medium pH was initially adjusted to three different levels (6.8, 8.5, and 8.8). After the initial enrichment phase (approximately 3 months), cultures at all three pH levels exhibited stable nitrite consumption coupled with nitrate production. The NOB strain enriched at pH 8.5 was first isolated, and the media formulation for purifying this culture was adjusted by adding (per liter) 10 mL HEPES (1M pH 7.5), 1 mL NaHCO_3_ (1 M), and 1 mL NaNO_2_ (1 M), omitting the vitamins and adjusting pH to 7.5. Subsequent 16S rRNA gene amplicon sequencing revealed that the same strain was also enriched at the other two pH levels (6.8 and 8.8). Therefore, we focused our detailed physiological and genomic characterization on the pH 8.5 culture. The enrichment of this strain across multiple pH conditions is consistent with its broad pH adaptation range (see Results for details).

Cultures were maintained in 100 mL volumes in acid-washed and autoclaved 250 mL GL45 bottles at 20°C, without shaking, in the dark. This initial temperature was selected to match the bioreactor conditions and to remain close to field-relevant temperatures. Activity was tracked by measuring NO_2_^−^ and NO_3_^−^ concentrations.

### Growth experiments

Growth experiments were conducted in at least triplicate using 100 mL cultures, each inoculated at 2% (vol/vol) with early stationary-phase maintenance cultures. Approximately 0.75–1 mL of the culture was aseptically sampled at regular intervals for cell counts and chemical analysis. Temperature optima were assessed across a range of 5°C–37°C using media at the original pH of 8.5. To determine pH optima, media were prepared from pH 5.0 to 9.0 in 0.5-unit increments, with MES used as the buffer below pH 7.0 in place of HEPES. After identifying pH 7.5 as optimal for the isolated strain, all subsequent experiments were performed at this pH. Incubations for pH, NaNO₂, NaNO₃, and NaCl tolerance experiments were conducted at 25°C.

For comparison, *N. vulgaris* strain-Z (DSM 10236) and *N. winogradskyi* Nb-255 (DSM 10237) were obtained from Leibniz Institute German Collection of Microorganisms and Cell Cultures (DSMZ) and were maintained in the minimal HEPES-buffered media described above with 1 mM NaNO_2_ at pH 7.5 and 25°C. All growth experiments were conducted in the dark without agitation.

Culture purity was routinely verified by plating on R2A agar and confirming the absence of colony formation after 5 days of incubation at 30°C. Culture identity was further confirmed by Sanger sequencing of the 16S rRNA gene, ensuring a single, high-quality chromatogram with no mixed-base signals.

### Chemical measurements

NO_2_^−^ and NO_3_^−^ concentrations were measured either on a Gallery discrete analyzer using off-the-shelf Gallery reagents for NO_2_^−^ and total oxidizable nitrogen (TON) (Cat. No: 984369, 984370, and 984371, Thermo Scientific, Waltham, MA) or on a BioTek Synergy multi-mode microplate reader using Griess reagent, according to EPA method 353. For TON measurement, NO_3_^−^ was first reduced to NO_2_^−^ using either hydrazine or vanadium chloride, followed by the Griess reaction to quantify total NO_2_^−^ (25). NO_3_^−^ concentrations were calculated by subtracting the NO_2_^−^ from the TON concentration.

### Cell counting and growth rate estimation

Cell concentrations were obtained using a Guava easyCyte (Cytek Biosciences, Fremont, CA, USA) flow cytometer with SYBR Green fluorescent DNA staining (26). Samples were diluted in Tris-EDTA buffer to a final concentration of 10⁴–10⁶ cells/mL. SYBR stain stock solution (1:30 dilution) was added at 10 µL per milliliter of diluted sample, followed by incubation at 37°C for 30 min. The flow cytometer was operated at a medium flow rate (0.59 µL/s). Cells were gated based on green fluorescence, and data collection continued until either 5,000 events were recorded or 123 µL of the sample was processed, whichever occurred first. Flow cytometry counts were initially validated against direct microscopic counts to ensure accuracy. Growth rates were estimated from the slope of natural-log transformed cell counts vs. time during the exponential phase.

### Scanning electron microscopy (SEM)

Cell morphology was observed using a scanning electron microscope (SEM). Mid-exponential phase strain MLSD-S22 cells were fixed using 4% glutaraldehyde in 0.1 M phosphate buffer (pH 7.4) for overnight at 4°C. After fixation, samples were washed three times with 0.1 M phosphate buffer (10 min each), followed by three washes with distilled water (10 min each). Dehydration was carried out with a graded ethanol series (25%, 50%, 75%, 85%, and 95%) for 10 min each, followed by three washes in 100% ethanol (10 min each). Samples were then critical point-dried and metal-coated for SEM imaging. At each step, buffers or supernatants were removed by centrifugation at 5,000 rpm for 5 min. Each preparation was examined with a Zeiss NEON Field-Emission Scanning Electron Microscope (SEM)/Focused Ion Beam (FIB) at a magnification of 20,000 (Samuel Robert Noble Microscopy Laboratory, University of Oklahoma).

### Microrespirometry-based whole-cell kinetic measurements

Whole-cell kinetic measurements were conducted using a MicroRespiration System equipped with OX-MR O₂ microsensors (Unisense, Aarhus, Denmark). Cultures were grown to high cell densities by repeatedly replenishing NO₂⁻ after its depletion, resulting in approximately 5 mM of total NO₂^−^ oxidized. Cells were harvested by centrifugation in 50 mL Falcon tubes (10,000 × *g*, 20 min, 15°C). Pellets were resuspended in a reduced volume, transferred to 1.5 mL centrifuge tubes, and centrifuged again to remove residual media. The resulting cell pellet was resuspended in 1.4 mL of fresh nitrogen-free media and transferred to autoclaved 1.1 mL glass microrespirometry chambers containing glass stir bars. A sample was taken for cell counting before the experiment.

All measurements were performed in a circulating water bath maintained at 28°C. Microsensors were calibrated daily using a two-point calibration with N₂- and air-sparged water. Baseline O₂ consumption was established by allowing cultures to equilibrate for approximately 1 h until stable readings were obtained. Subsequently, a NaNO₂ solution was injected to achieve an initial concentration of ~150 µM NO₂^−^. O₂ consumption was monitored until it returned to baseline for approximately 30 min before repeating the injection. To assess O₂ affinities, a final injection of excess NO₂^−^ was added to induce O₂-limited conditions (≤0.3 µM, the detection limit of the Unisense OX-MR microsensor).

Apparent half-saturation constant (*K*_m(app)_) and maximum cell-specific rate (*V*_max_) were derived from the O₂ consumption traces using equation 1 below, where [S] represents the concentration of O_2_ or NO_2_^−^, depending on the substrate kinetics being assessed. Substrate consumption rates were calculated using a 60-s sliding-window average, and NO₂^−^ concentrations and consumption rates were inferred from the O₂ data based on the 2:1 stoichiometry of NO₂^−^ oxidation. The rates were normalized on a per-cell basis using cell counts. Michaelis–Menten parameters were fitted to equation 1 by minimizing the sum of squared differences between observed and modeled rates using Microsoft Excel’s Solver function. The quality of fit was further assessed visually with Eadie–Hofstee plots (27). Protein content per cell was estimated from SEM-based cell volume measurements and an empirically derived protein to cell volume relationship (28) and was used to convert cell-specific parameters to protein-based values.


(1) $$\frac{d\left[S\right]}{dt}=\frac{V_{max}[S]}{K_{m(app)}+[S]}$$

Specific affinity (*a*^o^) in units of l (g wet-cell)^−1^ h^−1^ was calculated from the protein-normalized *V*_max_ and *K*_m(app)_ using equation 2. The factor 5.7 g wet-cell weight per g protein was used to estimate wet weight from protein content (29, 30).


(2) $$a^{o}=\frac{V_{max}}{K_{m(app)}}$$

### Genome sequencing and analysis

Cells were harvested by centrifugation from 100 mL of 5 mM NO_2_^−^ grown culture. Cell pellets were lysed, and DNA was extracted using the *Quick*-DNA HMW MagBead Kit (Zymo Research, Irvine, California, USA). The library was prepared using the Rapid Barcoding Kit 96 V14 (Oxford Nanopore) and was sequenced using a MinION Mk1c nanopore long-read sequencer (Oxford Nanopore, Oxford, United Kingdom) at the Institute for Systems Biology in Seattle, Washington, USA. Base calling was performed using Dorado v0.5.2 with the super high accuracy model (dna_r10.4.1_e8.2_400bps_sup@v4.2.0) (Oxford Nanopore). A total of 477,072 reads were generated with a mean length of 3,898 bp.

Long reads were assembled and annotated using Flye v2.9.4 (31) and RASTtk (32) as implemented in KBase (33). Genomes of related organisms were retrieved from the NCBI RefSeq database. Genome average nucleotide identity (ANI) was determined using FastANI v0.1.3 (34) as implemented in KBase (33). Genome circle plots were created using Proksee (35). *Nos* (encoding nitrous oxide reductase), *nar* (encoding respiratory nitrate reductase), and *nxr* (encoding nitrite oxidoreductase) operons were extracted from GenBank files using a custom Python script and were visualized using clinker v0.0.31 (36). Maximum-likelihood phylogenetic trees were constructed using MEGA11 (37) using 100 bootstrap replications, default parameters, and MUSCLE (38) for multiple sequence alignment.

## RESULTS

*Nitrobacter vulgaris* strain MLSD-S22 was enriched from a nitrifying bioreactor inoculated with groundwater from a site contaminated with nitric acid (HNO_3_) and a mixture of heavy metals. After approximately 3 months of batch enrichment culturing with low NO_2_^-^ concentrations (0.1 mM), stable nitrite oxidation to nitrate was achieved. 16S rRNA amplicon sequencing indicated that *Nitrobacter* was the only NOB population in the enrichments and comprised <10% of the total microbial community. To further enrich the NOB population within the mixed community, the NO_2_^−^ concentration was increased from 0.1 to 1 mM during subsequent enrichment and purification steps. Increasing the NO_2_^−^ concentration along with continued endpoint dilutions led to progressive enrichment of the *N. vulgaris* population. Continued serial dilution and cultivation over the course of approximately 2 years ultimately yielded a pure culture free of heterotrophic contaminants with purity verified by 16S rRNA amplicon sequencing and plating on heterotrophic media (R2A and LB broth).

**Fig 1:**
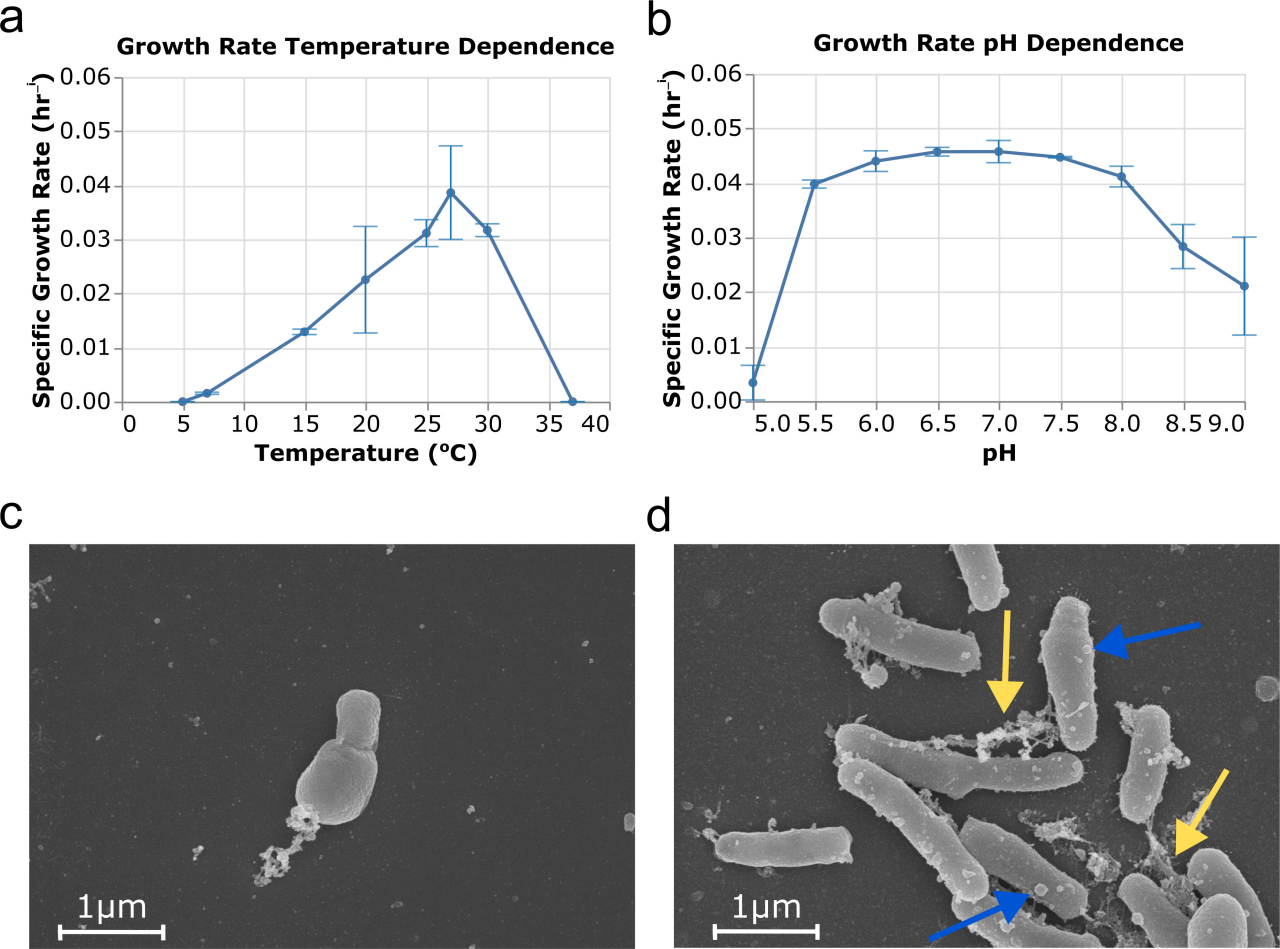
*N. vulgaris* strain MLSD-S22-specific growth rate dependence on temperature and pH, and SEM images. Specific growth rate dependence on temperature (**a**) and pH (**b**) with error bars showing standard deviation. Temperature dependence was determined at pH 8.5, and pH dependence was determined at 25°C. SEM images of strain MLSD-S22 showing budding-like cell division (**c**), and the presence of membrane vesicles (blue arrows) and extracellular polymeric substances (yellow arrows) (**d**).

**Fig 2:**
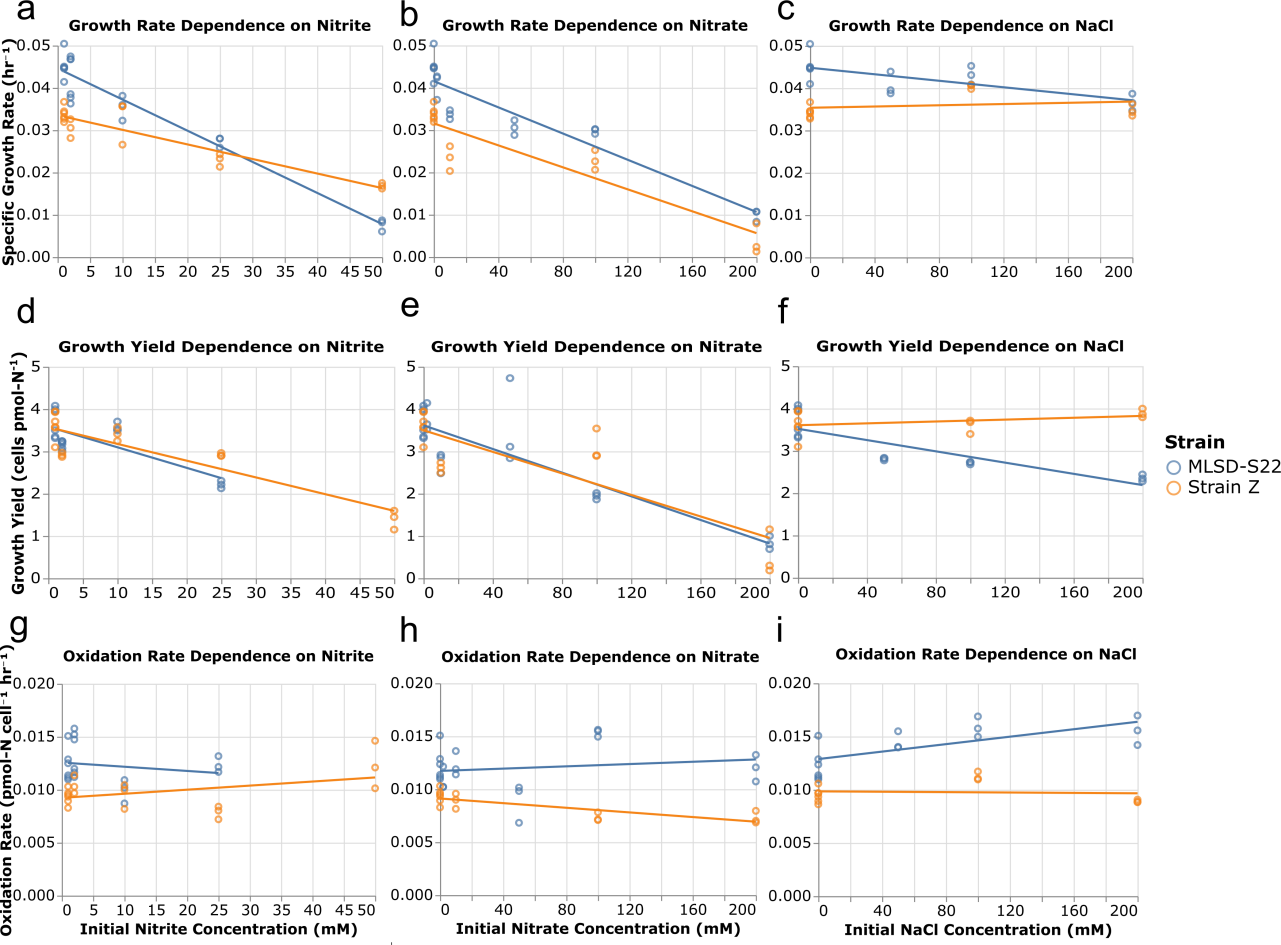
Growth inhibition by NO_2_^−^, NO_3_^−^, and NaCl in strain MLSD-S22 and strain Z. Specific growth rate dependence on initial NO_2_^−^ (**a**), NO_3_^−^ (**b**), and NaCl (**c**). Growth yield dependence on initial NO_2_^−^ (**d**), NO_3_^−^ (**e**), and NaCl (**f**). Cell-specific oxidation rate dependence on initial NO_2_^−^ (**g**), NO_3_^−^(**h**), and NaCl (**i**). Cell-specific oxidation rate values were derived from specific growth rate and yield values. Growth yield and oxidation rate are omitted for strain MLSD-S22 at 50 mM NO_2_^-^ due to incomplete substrate utilization. Linear regression parameters are listed in Tables S1 to S3.

Sanger sequencing of the 16S rRNA gene revealed 100% identity to existing *N. vulgaris* isolates (strains Z and Ab_1_). An ASV matching this sequence was detected in four field samples from a previous survey of the sample site, albeit at a low relative abundance (<0.5% of total 16S rRNA reads) (21). Optimal growth was observed between 25°C and 30°C, pH 6.0–8.0, and up to 1.17% salinity (Fig. 1a and b and Fig. 2c). SEM imaging showed cells as pleomorphic rods with an average length of 1.3 ± 0.3 µm and an average width of 0.4 ± 0.1 µm (Fig. 1c and d). Cell morphologies consistent with both budding-like division and binary fission were observed (Fig. 1c and d), a feature that has been reported for other *Nitrobacter* species (39). SEM images showed the presence of outer membrane vesicles and extracellular polymeric substances on cells (Fig. 1d).

To evaluate physiological variation within the species, we conducted comparative tests using both the isolate strain MLSD-S22 and the designated type strain Z. At 25°C and pH 7.5 on 1 mM of NO_2_^−^, strain MLSD-S22 had a growth rate of 0.045 ± 0.003 h^−1^ (doubling time of 15 ± 1 h), a yield of 3.7 ± 0.4 cells·pmol-N^−1^ (0.11 ± 0.04 mg-protein·mmol-N^−1^), and an oxidation rate of 0.012 ± 0.002 pmol-N·cell^−1^·h^−1^. Under the same conditions, we observed *N. vulgaris* strain Z to have a significantly lower (*P* < 0.001) growth rate of 0.034 ± 0.002 h^−1^ (doubling time of 21 ± 1 h), a similar yield of 3.6 ± 0.3 cells·pmol-N^−1^ (0.11 ± 0.04 mg-protein·mmol-N^−1^), and a significantly lower oxidation rate of 0.0093 ± 0.0007 pmol-N·cell^−1^·hr^−1^ (*P* < 0.01).

### Characterizing growth inhibition by elevated NO_2_^-^ and NO_3_^-^

Growth experiments were performed with strains MLSD-S22 and Z (DSM 10236) to characterize the inhibitory effects of increasing NO_2_^−^ and NO_3_^−^ concentrations on growth using Cl^−^ as a reference for osmotic stress. Originating from a high NO_3_^-^ setting, we hypothesized that *N. vulgaris* strain MLSD-S22 would have a higher tolerance to these stressors compared to strain Z.

The growth of both strains was more sensitive to elevated NO_2_^−^ than to increased NO_3_^−^ concentrations. Strain Z was less sensitive to high NO_2_^−^ concentrations compared to strain MLSD-S22, maintaining growth at 50 mM NO_2_^−^, while strain MLSD-S22 did not (Fig. S1). In both strains, specific growth rate decreased linearly with initial NO₂⁻ concentration; however, MLSD-S22 exhibited a significantly steeper decline (−0.00072 h⁻¹·mM NO₂^−^¹, R² = 0.76) compared to strain Z (−0.00034 h⁻¹·mM NO₂^−^¹, R² = 0.83; ANCOVA, *P* < 0.01) (Fig. 2a and Table S1). Growth yields also decreased at higher NO_2_^-^ concentrations at a rate of −0.049 cell·pmol-N^−1^·mM-NO_2_^−1^ for strain MLSD-S22 (R^2^ = 0.59) and −0.040 cell·pmol-N^−1^·mM-NO_2_^−1^ for strain Z (R^2^ = 0.79) (Fig. 2d and Table S1). Cell-specific oxidation rates remained relatively constant across NO_2_^−^ concentrations with slopes not significantly (*P* > 0.05) different from zero (Fig. 2g and Table S1).

Both strains were inhibited by NO_3_^−^; however, NO_3_^−^ inhibition was less severe than NO_2_^−^ with the two strains maintaining the ability to grow at concentrations up to 200 mM NO_3_^−^ (Fig. 2b; Fig. S2). Specific growth rate declined similarly in the two strains, with slopes of −0.000155 and −0.000130 h^−1^·mM-NO_3_^−1^ for strain MLSD-S22 and strain Z, respectively (Fig. 2b and Table S2). Growth yields also decreased at comparable rates (−0.0140 and −0.0127 cell·pmol-N^−1^·mM-NO_3_^−1^ for strain MLSD-S22 and strain Z, respectively; Fig. 2e and Table S2). In contrast, cell-specific oxidation rate was constant for strain MLSD-S22, while strain Z displayed a modest decrease with increasing NO_3_^−^ concentrations (ANCOVA, *P* < 0.05; Fig. 2h and Table S2). Overall, the specific growth rate and specific oxidation rates of strain MLSD-S22 were significantly higher than strain Z across the range of tested NO_3_^-^ concentrations (0-200 mM) (ANCOVA, *P* < 0.01; Fig. 2b and h).

Sodium cations were introduced while testing the impact of NO_3_^−^ and NO_2_^−^ on the growth of *N. vulgaris* strains. Therefore, NaCl was used to test salinity stress and distinguish osmotic effects from ion-specific toxicity. Both strains could grow in the presence of 200 mM NaCl (1.17% salinity or 11.7 Practical Salinity Unit, PSU) but showed no growth at 500 mM NaCl (2.92% salinity or 29.2 PSU) (Fig. S3). Overall, strain MLSD-S22 was more affected by increasing NaCl concentrations compared to strain Z. Strain Z’s growth rate, yield, and oxidation rate were constant from 0 to 200 mM NaCl, whereas strain MLSD-S22 showed greater decreases in specific growth rate and yield, along with a significant increase in oxidation rate, relative to strain Z (ANCOVA, *P* < 0.01; Fig. 2c, f and i, and Table S3). Still, the inhibition caused by NaCl was less pronounced compared to NO_3_^-^ and NO_2_^-^.

### Whole-cell kinetic measurements across *Nitrobacter* species

**Fig 3:**
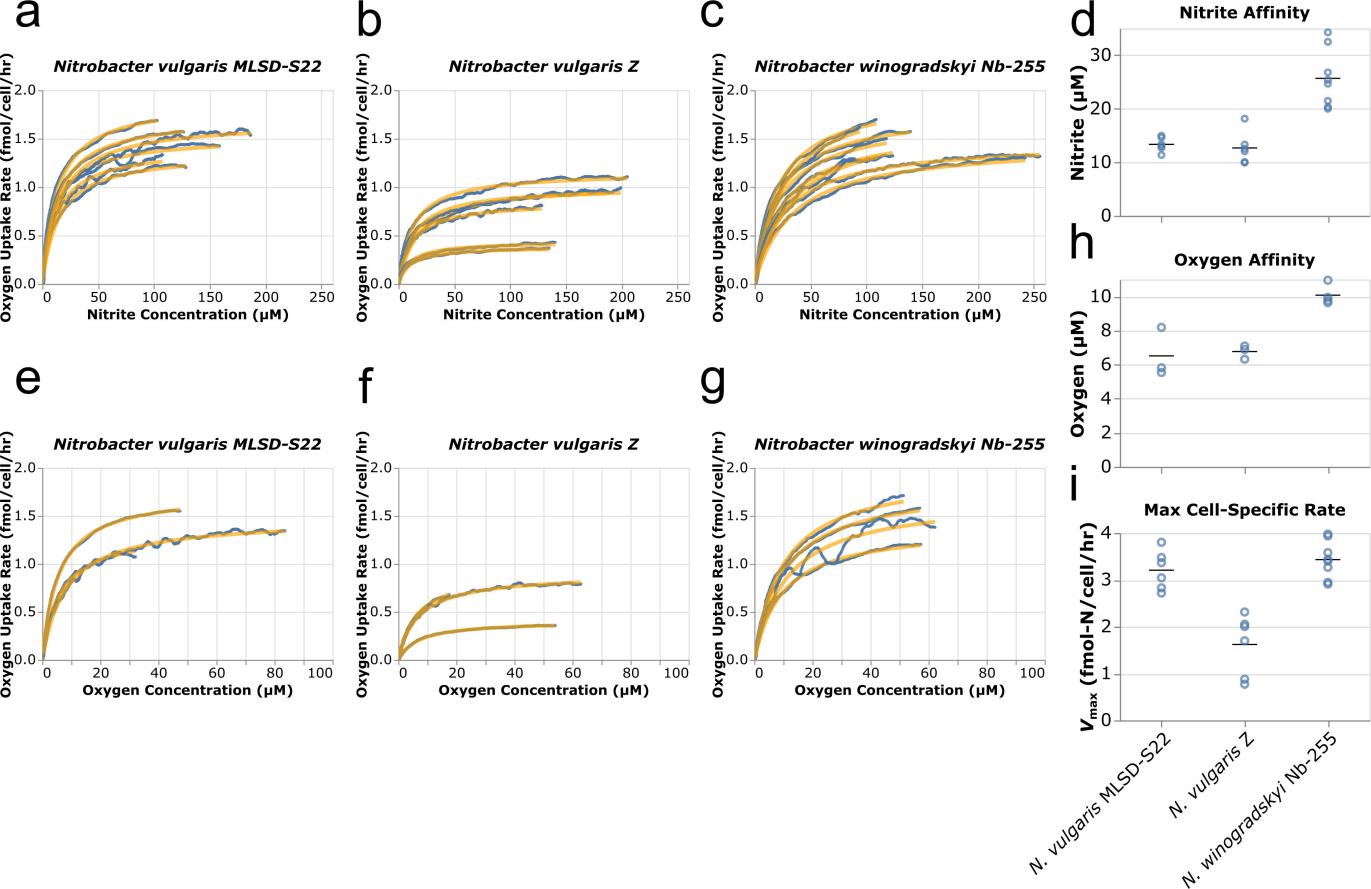
Nitrite oxidation kinetics across *Nitrobacter*. Traces of oxygen consumption rate vs. nitrite concentration for *N. vulgaris* strain MLSD-S22 (**a**), *N. vulgaris* strain Z (**b**), and *N. winogradskyi* strain Nb-255 (**c**). Blue lines show measured data and yellow lines represent the data fit based on Michaelis-Menten kinetics. Derived nitrite affinities (*K*_m(app)_) (**d**), oxygen affinities (*K*_m(app)_) (**h**), and max cell-specific oxidation rates (**i**). Analogous traces of oxygen consumption rate vs. oxygen concentration for *N. vulgaris* strain MLSD-S22 (**e**), *N. vulgaris* strain Z (**f**), and *N. winogradskyi* strain Nb-255 (**g**).

Whole-cell kinetic analysis of *N. vulgaris* strain MLSD-S22, *N. vulgaris* strain Z, and *N. winogradskyi* Nb-255 was conducted using microrespirometry, with the aim of comparing with previously reported data (17) and evaluating potential kinetic features underlying niche differentiation. NO_2_^−^-dependent O_2_ consumption followed Michaelis-Menten kinetics upon NO_2_^−^ injection in all NOB strains investigated here (Fig. 3; Fig. S4). Both *N. vulgaris* strains exhibited similar apparent half-saturation constants (*K*_m(app)_) for NO_2_^−^ (13.3 ± 1.3 µM (*n* = 6) and 12.7 ± 3.0 µM (*n* = 6) for strain MLSD-S22 and strain Z, respectively), while the *K*_m(app)_ for Nb-255 was significantly higher (25.7 ± 5.4 µM (*n* = 8)) (ANOVA and Tukey’s HSD: *P* < 0.0001) (Fig. 3d and Table 1). Similar trends were observed for O_2_ where the apparent half-saturation coefficients for the *N. vulgaris* strains (6.5 ± 1.5 µM (*n* = 3) and 6.8 ± 0.4 µM (*n* = 3) for strain MLSD-S22 and strain Z, respectively) were significantly lower than Nb-255 (10 ± 0.6 µM (*n* = 4)) (ANOVA and Tukey’s HSD: *P* < 0.01) (Fig. 3h and Table 1). Interestingly, *N. vulgaris* strain MLSD-S22 and *N. winogradskyi* Nb-255 displayed similar maximum cell specific NO_2_^−^ oxidation rates (3.2 ± 0.4 fmol-N·cell^−1^·h^−1^ [*n* = 6] and 3.4 ± 0.4 fmol-N·cell^−1^·h^−1^ [*n* = 8] for strain MLSD-S22 and strain Nb-255, respectively) that were significantly higher than *N. vulgaris* strain Z (1.6 ± 0.6 fmol-N·cell^−1^·h^−1^ [*n* = 6]) (ANOVA and Tukey’s HSD: *P* < 0.0001) (Fig. 3i and Table 1). Our measured *K*_m(app)_ values were generally lower than previously published values for *Nitrobacter* species, while *V*_max_ measurements were similar (Table S4) (17, 40–44).

**TABLE 1:** Kinetic parameters for tested strains derived from microrespirometry^*a*^

Organism	*K*_m(app)_ (µM NO_2_^-^)	*K*_m(app)_ (µM O_2_)	*V*_max_ (fmol-N cell^−1^ h^−1^)	*V*_max_ (µmol-N mg-protein^-1^ h^−1^)	*a*^o^ [(l (g wet-cell)^−1^ h^−1^]
*N. vulgaris* strain MLSD-S22	13.3 ± 1.3	6.5 ± 1.5	3.2 ± 0.4	105 ± 40	1,385 ± 545
*N. vulgaris* strain Z	12.7 ± 3.0	6.8 ± 0.4	1.6 ± 0.6	53 ± 30	732 ± 449
*N. winogradskyi* strain Nb-255	25.7 ± 5.4	10.1 ± 0.6	3.4 ± 0.4	112 ± 40	765 ± 317

^*a*^ Protein-based maximum oxidation rate (*V*_max_) and specific affinity (*a*^o^) were estimated from cell-specific rates, expected protein content, and a protein to cell weight conversion factor (details in methods).

### Strain MLSD-S22 genome characteristics and comparative analysis

A complete genome of *N. vulgaris* strain MLSD-S22 was obtained through Oxford Nanopore long-read sequencing. The genome consisted of a 4.18 Mbp chromosome (59.4% G + C) along with two plasmids of length 242 kbp and 164 kbp (58.5% and 61.1% G + C). The genome and plasmids had an average nucleotide identity (ANI) of 99.18% with *N. vulgaris* strain Ab_1_, 97.77% with *N. vulgaris* strain Z, 86.56% with *N. winogradskyi* strain Nb-255, and 84.9% with *N. hamburgensis* strain X14.

**Fig 4:**
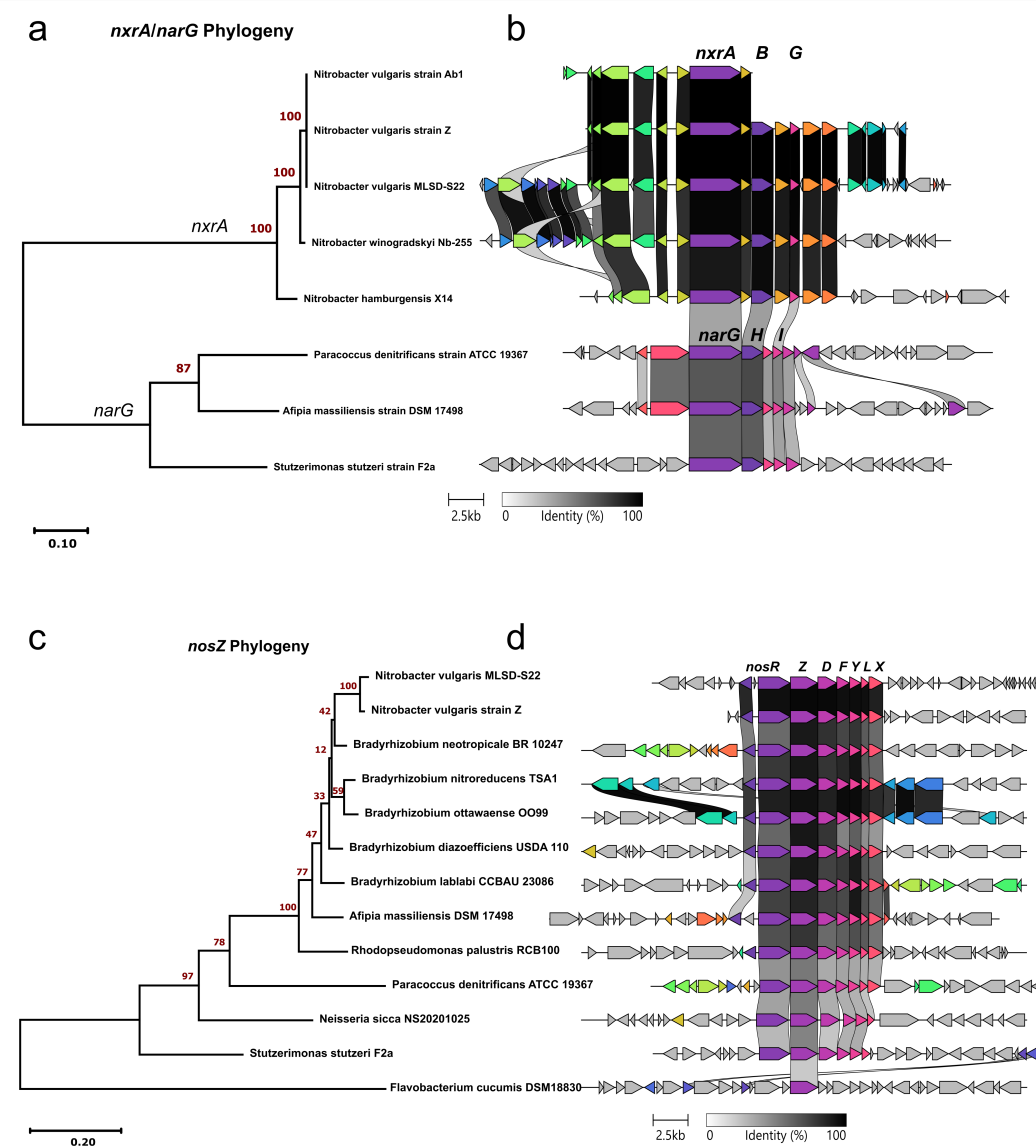
Phylogeny and operon structure of nitrite oxidation and nitrous oxide reduction. Maximum likelihood phylogenetic tree of *nxrA* and *narG* from *Nitrobacter* and related denitrifiers, respectively (**a**). Operon structure of *nxrA*/*narG* used in tree construction (**b**). Maximum likelihood phylogenetic tree of *nosZ* from *Nitrobacter vulgaris* and related denitrifiers (**c**). Nos operon structure for genes used in the tree (**d**). Bootstrap values shown at the nodes of the phylogenetic trees are based on 100 replicates. Colors and shaded links in (**b**) and (**d**) represent pairwise amino acid similarity. Interactive clinker-generated operon structure comparisons, which allow full exploration of gene annotations, are available in the project’s code repository (https://github.com/zflink/Nitrobacter_characterization/tree/main/Genomics/clinker_figures).

The strain MLSD-S22 genome contained a complete nitrite oxidoreductase (*nxr*) operon for nitrite oxidation, along with additional paralogs of *nxrA* and *nxrB*, as has been reported in other *Nitrobacter* genomes (45). The copy of *nxrA* within the complete operon was 100% identical to *N. vulgaris* strains Z and Ab1, 98.1% identical to *N. winogradskyi* strain Nb-255, and 91.7% identical to *N. hamburgensis* strain X14 (Fig. 4a). The overall operon structure was highly conserved across *Nitrobacter* species, with one notable difference: the absence of a glycosyltransferase protein upstream of the operon in *N. hamburgensis* (Fig. 4b). Additionally, the *nxrA* genes from *Nitrobacter* shared 47% identity with the respiratory nitrate reductase (*narG*) found in related denitrifiers (Fig. 4a). There was also partial conservation in operon structure between *nxrABG* and *narGHI* across these genomes (Fig. 4b).

Surprisingly, the *N. vulgaris* strain MLSD-S22 genome contained a putative complete operon for N_2_O reduction (*nosRZDFYL*), a feature recently identified in *N. vulgaris* strain Z (46) but absent in other *Nitrobacter* genomes. Analysis of the *nosZ* (encoding the active-site-containing subunit of nitrous oxide reductase) amino acid sequence revealed that the two *N. vulgaris* strains were highly similar (97.4%), with the next closest BLAST matches (82%–91% identity) found in *Bradyrhizobium, Afipia*, and *Rhodopseudomonas* species, all members of the *Nitrobacteraceae* family with clade-I type *nosZ* for which N_2_O reductase activity has been experimentally verified (47–51) (Fig. 4c). *nosZ* sequences from outside *Alphaproteobacteria* exhibited decreasing similarity, with the lowest identity (32%) observed in the clade-II *nosZ* from *Flavobacterium cucumis* (phylum *Bacteroidota*) (52). The *nos* operon displayed a high degree of conservation, with its gene order largely preserved across sampled genomes from *Pseudomonadota* (Fig. 4d). However, despite the high sequence similarity of *nosZ* to those of experimentally verified N_2_O reducers and the presence of a complete putative N_2_O reductase operon, no N_2_O reduction activity was detected for strain MLSD-S22 under the conditions tested (see Supplementary Note for experimental details).

For additional nitrogen metabolism, the strain MLSD-S22 genome contained an assimilatory (*nirBD*) and dissimilatory NO (nitric oxide)-forming (*nirK*) nitrite reductase genes. The genome did not contain any other genes for denitrification (e.g., respiratory nitrate reductase [*nar*], periplasmic nitrate reductase [*nap*], and nitric oxide reductase [*nor*]), which is consistent with other *Nitrobacter*. In terms of autotrophic carbon metabolism, the genome encoded a complete Calvin-Benson-Bassham (CBB) cycle, including a ribulose bisphosphate carboxylase (RuBisCo), which was alongside genes necessary for carboxysome formation. The genome encoded nearly complete pathways for glycolysis, the citrate cycle, and the glyoxylate cycle. Additionally, it harbored the capacity for the utilization of acetate, pyruvate, and phenylacetate (Fig. S5a), along with genes involved in the synthesis and degradation of poly-hydroxyalkanoates and polyphosphate, which serve as storage and energy polymers. The genome harbored both cobalamin-independent and -dependent methionine synthase genes (*metE* and *metH*), but no pathway for *de novo* cobalamin biosynthesis. The presence of *metE* is consistent with our physiological results showing that strain MLSD-S22 can grow in medium without vitamin supplementation. Additionally, genomic analysis of strain MLSD-S22 identified canonical osmotic-stress systems, including trehalose biosynthesis genes for compatible-solute production and an NhaP-type Na^+^/H^+^ antiporter that facilitates Na^+^ efflux during salt stress.

The two plasmids harbored distinct sets of genes related to stress resistance, carbon metabolism, and conjugation. The 242 kbp plasmid encoded genes associated with glycogen metabolism, the type IV secretion system (vir-type), heat shock proteins, and chemotaxis (Fig. S5b). In contrast, the 164 kbp plasmid contained multiple genes involved in heavy metal efflux (especially copper), organic carbon metabolism (including xylose, acetate, and lactate utilization), transposases, and a type IV secretion system (trb-type) (Fig. S5c).

## DISCUSSION

We isolated an *N. vulgaris* strain originating from a contaminated subsurface and characterized its ecophysiology and genomic potential. Given the strain was isolated from an environment with high NO_3_^−^ concentrations and low pH, we were interested in identifying adaptive features of the new strain that enabled it to continue oxidizing NO_2_^−^ under relevant environmental conditions. We found that strain MLSD-S22 exhibited a broad pH tolerance and was capable of maintaining active NO_2_^−^ oxidation down to pH 5.5, supporting its ecological persistence and activity in the subsurface environment from which it was isolated (pH 6.6–6.7). We hypothesized that it would have a higher tolerance for NO_3_^−^ compared to other strains. Contrary to our hypothesis, the tested *N. vulgaris* strains exhibited similar growth responses to increasing NO_3_^−^, NO_2_^−^, and NaCl concentrations. In fact, strain MLSD-S22 was more sensitive to NO_2_^-^ and NaCl than the reference strain. Based on our results here, the highest NO_3_^−^ concentrations measured at the Oak Ridge site would inhibit the lithotrophic growth of strain MLSD-S22. Whether or not this NOB shifted its metabolism toward heterotrophic denitrification under elevated NO_3_^−^ conditions would be interesting to explore.

Data on *Nitrobacter* growth inhibition by these ions are limited. One study measured *N. winogradskyi* strain agilis activity based on oxygen consumption and reported higher tolerance levels than those observed here, with more than 50% activity maintained at 100 mM NO_2_^−^, ~20% activity at 250 mM NO_3_^−^, and over 50% activity at 500 mM NaCl (41). The study focused solely on oxygen consumption rather than growth, which could explain the higher values.

Our inhibition experiments revealed constant cell-specific oxidation rates in contrast to decreasing growth rate and yield with increasing ion concentration. This suggests that *N. vulgaris* can maintain stable NO_2_^-^ turnover across environmental gradients; however, increased maintenance energy demands diverted ATP away from growth to sustain cellular homeostasis. NO_2_^−^ is particularly toxic due to its role in generating reactive oxygen and nitrogen species, requiring cells to increase their stress response at elevated concentrations (53, 54). Additionally, NO_2_^-^ may function as a protonophore (54). Our observations indicated that NO_3_^−^ was less inhibitory than NO_2_^−^ but more inhibitory than NaCl, aligning with previous reports on *Nitrobacter* and sulfate-reducing bacteria (41, 55, 56). Thus, the inhibition caused by NO_3_^−^ must be due to more than osmotic stress. In sulfate-reducing bacteria, the transcriptomic response to NO_3_^−^ stress resembled that induced by NO_2_^-^ (53, 56), hinting at potential shared pathways of cellular disruption. However, we did not observe any increase in NO_2_^−^ concentrations during our experiments with elevated NO_3_^−^, which would suggest that disruption was occurring via NO_2_^−^. Thus, additional mechanisms, such as interference with NO_2_^−^/NO_3_^−^ transport (57), thermodynamic limitation under high NO_3_^-^ concentrations, or interactions between NO_3_^-^ and enzyme metal centers, may be at play.

Substrate affinity is a key niche differentiating factor for NOB (17), and there is ample evidence from wastewater showing that *Nitrobacter* are enriched under high NO_2_^−^ concentrations whereas *Nitrospira* are enriched under low NO_2_^−^ concentrations (18, 44, 58). However, our measured *K*_m(app)_ values for NO_2_^−^ were lower than many reported values for *Nitrobacter* species (Table S4) (17, 41, 44, 59), but comparable to those of an acidophilic enrichment (14 ± 2 µM) (43), strain JJSN (11.3 ± 2.7 µM) (57), and *N. vulgaris* strain CN101 (29.0 µM) (60), which was enriched from an oligotrophic forest soil. They were also similar to values reported for *N. winogradskyi* strain Nb-255 grown at 1 mM NO₂^−^ (25.9 ± 5.2 µM) (57). These comparisons indicate that some *Nitrobacter* can exhibit high substrate affinities under certain conditions.

Differences in culture conditions may have contributed to these results: our strain was initially enriched with only 0.1 mM NO_2_^−^ and was subsequently isolated and maintained in media containing 1 mM NO_2_^−^, which may affect cellular proteome allocation under relatively low nutrient concentrations. In contrast, cultures routinely grown with 10 mM NO_2_^−^ potentially lead to higher *K*_m(app)_ values and thus lower substrate affinities. A recent study showed that *Nitrobacter* cultures grown with 10 mM NO₂⁻ had significantly higher *K*_m(app)_ values than those grown with 1 mM NO₂⁻, and that cultures tested at pH 7.5 had higher *K*_m(app)_ values than at pH 5.5 (57). Kinetic adaptation based on culture conditions has been observed in *Nitrobacter* grown with or without acetate (40), at different oxygen concentrations (42, 59), and in ammonia-oxidizing microorganisms maintained on ammonia vs. urea (30). Further investigation into the effects of culture conditions and cellular preconditioning on kinetic properties is warranted based on these observations. A related methodological limitation is that our kinetic parameters were derived from single-injection assays; although multiple-injection approaches can reduce parameter uncertainty, single-injection designs are commonly used in microbial kinetics, and our measurements were internally consistent across replicates and conditions, providing a reliable baseline for comparative analyses.

*Nitrobacter* possesses an aa_3_-type cytochrome c oxidase, an enzyme with affinities reported in the range of 0.97–5.0 µM O_2_ (61). Our measurements (6.5–10.1 µM O_2_) are slightly above this range and are comparable to *Nitrosomonas europaea*, an ammonia-oxidizing bacterium with a similar cytochrome c oxidase (42, 59), and slightly above *N. japonica*, a NOB harboring a *bd-like* terminal oxidase (62). Previous reports of *Nitrobacter* O_2_ affinity are considerably variable, ranging from 5.2 to 175 µM O_2_ (42, 43), and our measurements fall at the lower end of this reported range. This variability could be caused in part by the presence of NO, which *Nitrobacter* can produce via a NirK NO_2_^−^ reductase. NO can increase the *K*_m(app)_ by interacting with cytochrome c oxidases (63). In *Nitrobacter* specifically, NO may direct electron flow away from O_2_ reduction toward CO_2_ fixation and poly-hydroxybutyrate synthesis (64).

Using long-read DNA sequencing, we assembled a closed genome for *N. vulgaris*, which may aid in refining the draft genomes of strains Z and Ab_1_ (46, 65). The genome exhibited high similarity to existing isolates, with notable exceptions. Most strikingly, we identified a putative nitrous oxide reductase (*nos*) operon, previously reported only in the strain Z draft genome (46), but absent in other *Nitrobacter* species. Phylogenetic and operon analysis revealed a relationship to other *Alphaproteobacteria nosZ* with experimentally verified activity (49–51), although the two *Nitrobacter* sequences were clearly divergent. Although the genome encodes a complete N₂O reduction operon, N₂O reduction activity was not detected under the conditions tested with acetate, pyruvate, and NO_2_^−^ provided as electron donors, indicating that any contribution to N_2_O reduction may be limited or dependent on specific environmental factors not captured here (Supplementary Note). This raises questions about the role of N_2_O reduction in *Nitrobacter* physiology. *Nitrobacter* generally harbor a copper-containing nitrite reductase (NirK) that converts NO_2_^−^ to NO. Indeed, previous studies have demonstrated NirK expression and the formation of NO and N_2_O in *Nitrobacter* cultures (10, 64, 66). While no reduction was observed under the conditions tested here, it is possible that under certain conditions, *Nitrobacter* function as heterotrophic denitrifiers, utilizing N_2_O as an electron acceptor in specific environments with high N_2_O fluxes, such as the Oak Ridge site (67). There may be a preconditioning effect in which cells must be exposed to organic carbon before shifting to a mixotrophic or heterotrophic mode (68, 69). In our experiments, cultures were grown under chemolithoautotrophic conditions and thus may not have been primed for heterotrophic processes such as N_2_O reduction. Future work should more directly assess these preconditioning effects. Just as preconditioning to different NO₂⁻ concentrations influenced substrate affinity, prior exposure to organic carbon may similarly affect mixotrophic activity (69).

Additionally, we identified two plasmids in the *N. vulgaris* genome containing genes related to type-IV secretion systems, carbon metabolism, and heavy metal resistance—which is notable, given the high levels of heavy metal contamination at the Oak Ridge site. Similarly, three plasmids were identified in *N. hamburgensis* X14, with genes related to central metabolism, conjugation, and autotrophy (45). However, the latter was absent in *N. vulgaris* plasmids. Given the prevalence of plasmids in *Nitrobacter* and related bacteria such as *Bradyrhizobium* (70, 71), genetic exchange may be common among these organisms, playing a key role in their evolution and adaptation. This may have contributed to either the acquisition of the *nos* operon in *N. vulgaris* or its loss in other *Nitrobacter* species.

The kinetic and regression parameters derived from this study will be valuable for process modelers studying ecosystems where NOB shape local biogeochemistry, such as wastewater treatment (4, 72), marine oxygen minimum zones (73–75), wetlands (76), and agricultural soils. The biogeochemical models utilized to study these environments rely on isolate kinetic parameters to define the competition for NO_2_^−^ between defined functional guilds—denitrifiers, anammox bacteria, and other NOB genera. Our results suggest that some *Nitrobacter* may have a higher affinity for NO_2_^−^ than previously thought and may even overlap with some *Nitrospira* NOB (17, 57, 62). This will enhance model accuracy and help explain observed differences in the abundance of NOB lineages. Future research should focus on the regulation and diversity of their mixotrophic metabolism, particularly investigating whether *N. vulgaris* strains can perform N_2_O reduction under certain conditions. Furthermore, elucidating the role of NirK and NO in modulating electron flow and whole-cell affinities to O_2_ and NO_2_^−^ will provide deeper insights into nitrifier substrate preference and niche differentiation under environmentally relevant conditions.

## Data Availability

Project data and code are available from the following GitHub repository: https://github.com/zflink/Nitrobacter_characterization. The raw reads and genome assembly are available from the NCBI database under the BioProject accession number PRJN1298934.

## SUPPLEMENTAL MATERIAL

Supplemental material aem.00130-26-s0001.docx Supplemental methods, Tables S1 to S4, and Fig. S1 to S7.

ASM does not own the copyrights to Supplemental Material that may be linked to, or accessed through, an article. The authors have granted ASM a non-exclusive, world-wide license to publish the Supplemental Material files. Please contact the corresponding author directly for reuse.
