# Supplementary material for: Comparative physiological and genomic characterization of a novel Nitrobacter vulgaris strain from a nitrate-contaminated subsurface
Source: Appl Environ Microbiol. 2026 Mar 24;92(4):e00130-26. doi: 10.1128/aem.00130-26 (PMC13101473; doi:10.1128/aem.00130-26)
Supplement: Supplemental material — Supplemental methods, Tables S1 to S4, and Fig. S1 to S7. [file aem.00130-26-s0001.docx]

SUPPLEMENTARY INFORMATION

TITLE: Comparative physiological and genomic characterization of a novel *Nitrobacter vulgaris* strain from a nitrate-contaminated subsurface

SHORT TITLE: Physiological and genomic characterization of a *Nitrobacter vulgaris* strain

**Zachary Flinkstrom^1^, Kristopher A. Hunt^1,2^, Britt Abrahamson^1^, Pierce Harvell^3^, Xiangpeng Li^3^, James Wilson^2^, Zachary S. Cooper^2^, Jacob J. Valenzuela^2^, Nitin S. Baliga^2^, David A. Stahl^1^, Wei Qin^3,4^*, Mari-Karoliina H. Winkler^1^**

^1^ Department of Civil and Environmental Engineering, University of Washington, Seattle, WA, USA

^2^ Institute of Systems Biology, Seattle, WA, USA

^3^ School of Biological Sciences, Institute for Environmental Genomics, University of Oklahoma, Norman, OK, USA

4 Department of Microbiology, School of Molecular and Cellular Biology, Carl R. Woese Institute for Genomic Biology, University of Illinois Urbana-Champaign, Urbana, IL, USA

*Correspondence to: Wei Qin

**Supplementary Note**

Given the presence of the nitrous oxide (N_2_O) reduction operon in the genome, a brief experiment was conducted to assess whether strain MLSD-S22 was capable of reducing N₂O in the presence of various potential electron donors. Cultures were first grown aerobically with 1 mM NO₂⁻. Ten milliliters of fully grown culture (approximately 2x10^6^ cells/mL) was then transferred to Balch-style tubes, sealed with chlorobutyl rubber stoppers, and crimped with aluminum seals. The headspace was flushed with N₂ gas for 10 min, followed by injection of 1 mL of pure N₂O gas and addition of the following potential electron donors (final concentrations in parentheses): acetate (0.5 mM), pyruvate (0.5 mM), NO₂⁻ (5 mM), a combination of acetate (0.5 mM) and NO₂⁻ (5 mM), or no electron donor (control).

To monitor N₂O consumption, 0.1 mL of headspace gas was sampled and analyzed by gas chromatography (SRI 8610C, SRI Instruments, Torrance, CA, USA) equipped with a 1.83 m Haysep D column and a thermal conductivity detector (TCD). N₂ was used as the carrier gas at 17 psi, with oven and detector temperatures maintained at 70°C and 100°C, respectively. Acetate concentrations were determined by ion chromatography (IC) on a Dionex ICS 5000+ equipped with IonPac AS11-HC analytical and AS11 guard columns at 30°C. The eluent consisted of generated KOH (Dionex EG-5 and EGC III KOH, Thermo Fisher Scientific) at a flow rate of 1.5 mL min⁻¹. The KOH concentration was increased from 1 to 60 mM according to the following gradient: (i) 1 mM for 7 min, (ii) 1.56 mM min⁻¹ for 9 min, (iii) 1.67 mM min⁻¹ for 9 min, and (iv) 3.75 mM min⁻¹ for 8 min, then held at 60 mM for 90 s.

Little to no N₂O reduction was observed under any of the tested conditions after 300–500 h of incubation (Fig. S7). Although a slight declining trend in N₂O concentration was noted, it was within the analytical variability and could be attributed to loss of overpressure from repeated headspace sampling. The viability of cultures after N₂ sparging was confirmed by injecting O₂ and NO₂⁻ into parallel tubes, which showed robust aerobic NO₂⁻ oxidation activity (Fig. S7f). Measurement of acetate concentrations at the beginning and end of the 550 h incubation period indicated that only 56 µM of the supplied 500 µM acetate was consumed in the acetate-only condition, whereas 112 µM was consumed when both acetate and NO₂⁻ were provided, potentially consuming 10% of the provided N_2_O. Despite this additional acetate consumption, no corresponding increase in N₂O reduction was observed, as would be expected if the processes were coupled.

In conclusion, strain *N. vulgaris* strain MLSD-S22 showed no detectable N₂O reduction activity under the tested conditions.

**Supplementary tables**

**Table S1 – Linear regression parameters from NO_2_^-^ growth inhibition.** Growth rate is in units of hr^-1^, yield is in units of cells·pmol-N^-1^, and oxidation rate is in pmol-N·cell^-1^·hr^-1^. Slope is in units of growth rate/yield/oxidation rate per initial mM NO_2_^-^ added.

|  | Slope (per mM NO_2_^-^) | Intercept | R-value | p-value | Stderr of slope | Stderr of intercept | R^2^ |
| --- | --- | --- | --- | --- | --- | --- | --- |
| Strain MLSD-S22 Growth Rate | -0.000719 | 0.044520 | -0.869148 | 2.84E-06 | 0.000102 | 0.001132 | 0.755 |
| Strain MLSD-S22 Yield | -0.048535 | 3.582753 | -0.766649 | 2.06E-04 | 0.010162 | 0.112472 | 0.588 |
| Strain MLSD-S22 Oxidation rate | -0.000040 | 0.012563 | -0.187204 | 4.57E-01 | 0.000052 | 0.000574 | 0.035 |
| Strain Z Growth Rate | -0.000344 | 0.033526 | -0.913055 | 1.25E-07 | 0.000038 | 0.000892 | 0.834 |
| Strain Z Yield | -0.039560 | 3.572929 | -0.891061 | 7.05E-07 | 0.005038 | 0.116903 | 0.794 |
| Strain Z Oxidation Rate | 0.000038 | 0.009234 | 0.412730 | 8.87E-02 | 0.000021 | 0.000492 | 0.170 |

**Table S2 – Linear regression parameters from NO_3_^-^ growth inhibition.** Growth rate is in units of hr^-1^, yield is in units of cells·pmol-N^-1^, and oxidation rate is in pmol-N·cell^-1^·hr^-1^. Slope is in units of growth rate/yield/oxidation rate per initial mM NO_3_^-^ added.

|  | Slope (per mM NO_3_^-^) | Intercept | R-value | p-value | Stderr of slope | Stderr of intercept | R^2^ |
| --- | --- | --- | --- | --- | --- | --- | --- |
| Strain MLSD-S22 Growth Rate | -0.000155 | 0.041560 | -0.935023 | 5.34E-10 | 0.000013 | 0.001166 | 0.874 |
| Strain MLSD-S22 Yield | -0.013969 | 3.615253 | -0.871745 | 2.65E-07 | 0.001801 | 0.156137 | 0.760 |
| Strain MLSD-S22 Oxidation Rate | 0.000005 | 0.011731 | 0.177631 | 4.41E-01 | 0.000007 | 0.000595 | 0.032 |
| Strain Z Growth Rate | -0.000130 | 0.031580 | -0.914525 | 1.82E-06 | 0.000016 | 0.001592 | 0.836 |
| Strain Z Yield | -0.012719 | 3.497591 | -0.844224 | 7.55E-05 | 0.002240 | 0.224182 | 0.713 |
| Strain Z Oxidation Rate | -0.000011 | 0.009125 | -0.794495 | 4.03E-04 | 0.000002 | 0.000232 | 0.631 |

**Table S3 – Linear regression parameters from NaCl growth inhibition.** Growth rate is in units of hr^-1^, yield is in units of cells·pmol-N^-1^, and oxidation rate is in pmol-N·cell^-1^·hr^-1^. Slope is in units of growth rate/yield/oxidation rate per initial mM NaCl added.

|  | Slope (per mM NaCl) | Intercept | R-value | p-value | Stderr of slope | Stderr of intercept | R^2^ |
| --- | --- | --- | --- | --- | --- | --- | --- |
| Strain MLSD-S22 Growth Rate | -0.000038 | 0.044814 | -0.731544 | 1.94E-03 | 0.000010 | 0.001017 | 0.535 |
| Strain MLSD-S22 Yield | -0.006648 | 3.520165 | -0.853448 | 5.20E-05 | 0.001126 | 0.115374 | 0.728 |
| Strain MLSD-S22 Oxidation Rate | 0.000018 | 0.012883 | 0.683254 | 4.99E-03 | 0.000005 | 0.000532 | 0.467 |
| Strain Z Growth Rate | 0.000007 | 0.035363 | 0.215836 | 5.00E-01 | 0.000010 | 0.001169 | 0.047 |
| Strain Z Yield | 0.001100 | 3.605598 | 0.371402 | 2.35E-01 | 0.000870 | 0.097227 | 0.138 |
| Strain Z Oxidation Rate | -0.000001 | 0.009848 | -0.075658 | 8.15E-01 | 0.000004 | 0.000423 | 0.006 |

**Table S4 – Kinetic parameters for tested strains derived from microrespirometry compared to published values.** Protein-based maximum oxidation rate (*V*_max_) and specific affinity (*a*^o^) were estimated from cell-specific rates, expected protein content, and a protein to cell weight conversion factor (details in Methods).

| Organism and reference | *K*_m(app)_ (µM NO_2_^-^) | *K*_m(app)_ (µM O_2_) | *V*_max_ (fmol-N cell^-1^ hr^-1^) | *V*_max_ (µmol-N mg-protein^-1^ hr^-1^) | *a*^o^ ((l (g wet-cell)^−1^ hr^−1^) |
| --- | --- | --- | --- | --- | --- |
| *N. vulgaris* strain MLSD-S22 (This study) | 13.3 ± 1.3 | 6.5 ± 1.5 | 3.2 ± 0.4 | 105 ± 40 | 1385 ± 545 |
| *N. vulgaris* strain Z (This study) | 12.7 ± 3.0 | 6.8 ± 0.4 | 1.6 ± 0.6 | 53 ± 30 | 732 ± 449 |
| (1)*N. winogradskyi* strain Nb-255 (This study) | 25.7 ± 5.4 | 10.1 ± 0.6 | 3.4 ± 0.4 | 112 ± 40 | 765 ± 317 |
| *N. vulgaris* strain CN101 (1) | 25.3 and 32.6 (n = 2) | N/A | N/A | 79.4 and 78.1 (n = 2) | N/A |
| *Candidatus*Nitrobacter acidaffinis strain JJSN (2) | 11.3 ± 2.7 | N/A | N/A | 37.2 ± 1.4 | 377.5 ± 86.8 |
| *N. winogradskyi* strain Nb-255 (3) | 44 ± 23 | N/A | 2.7 ± 1.2 | N/A | N/A |
| *N. winogradskyi* strain Nb-255 (grown at 1 mM NO_2_^-^ and pH 7.5) (2) | 25.9 ± 5.2 | N/A | N/A | 68.8 ± 2.5 | 440.5 ± 167.5 |
| *N. winogradskyi* strain Nb-255 (grown at 10 mM NO_2_^-^ and pH 7.5) (2) | 388.0 ± 52.2 | N/A | N/A | 43.7 ± 1.3 | 14.4 ± 2.3 |
| *N.vulgaris* strain Ab_1_ (4) | 49 ± 11 | N/A | N/A | 164 ± 9 | 587 ± 136 |
| *N. winogradskyi* strain Engel (4) | 309 ± 92 | N/A | N/A | 78 ± 5 | 44 ± 13 |
| *N. winogradskyi* strain Agilis (5) | 406 ± 147 | 20 | N/A | N/A | N/A |
| *N. hamburgensis* strain X14 (4) | 544 ± 55 | N/A | N/A | 64 ± 1 | 21 ± 2 |
| *N. hamburgensis* strain X14(6) | 933 ± 234 | 57 ± 50. | 5.1 ± 1.5 | N/A | N/A |
| *N. hamburgensis* strain X14 (3) | 656 ± 435 | N/A | 1.8 ± 1.1 | N/A | N/A |
| Acidophilic *Nitrobacter spp* (enrichment)(7) | 14 ± 2 | 184 ± 37 | N/A | N/A | N/A |
| *Nitrobacter* enrichment (8) | 89 ± 6 | 13 ± 3 | N/A | N/A | N/A |

**Supplementary figures**
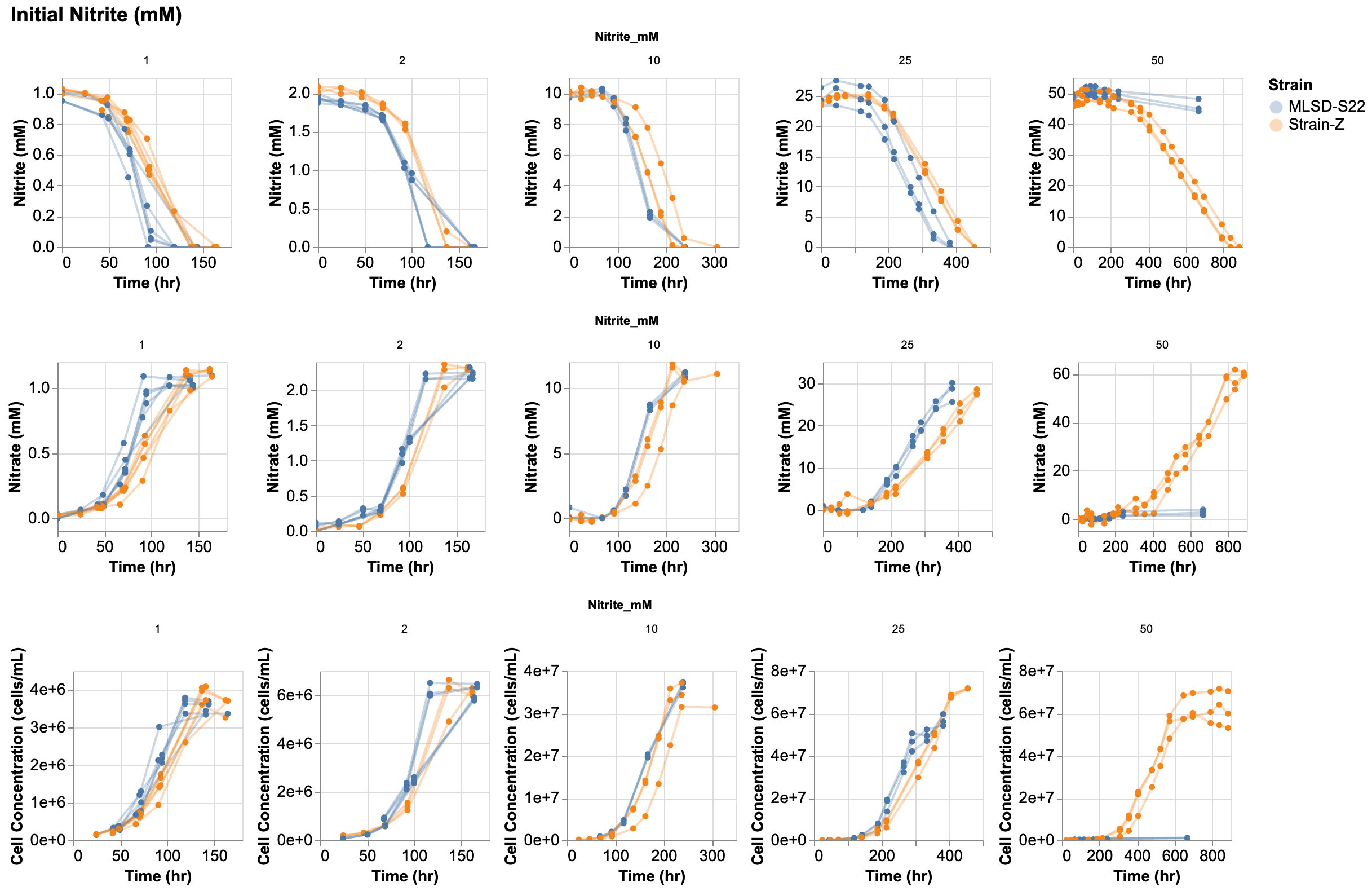


**Figure S1 – Growth curves for NO_2_^-^ inhibition experiments.** NO_2_^-^ consumption (top row), NO_3_^-^ accumulation (middle row), and cell counts (bottom row). Each column represents a different initial concentration of NO_2_^-^.


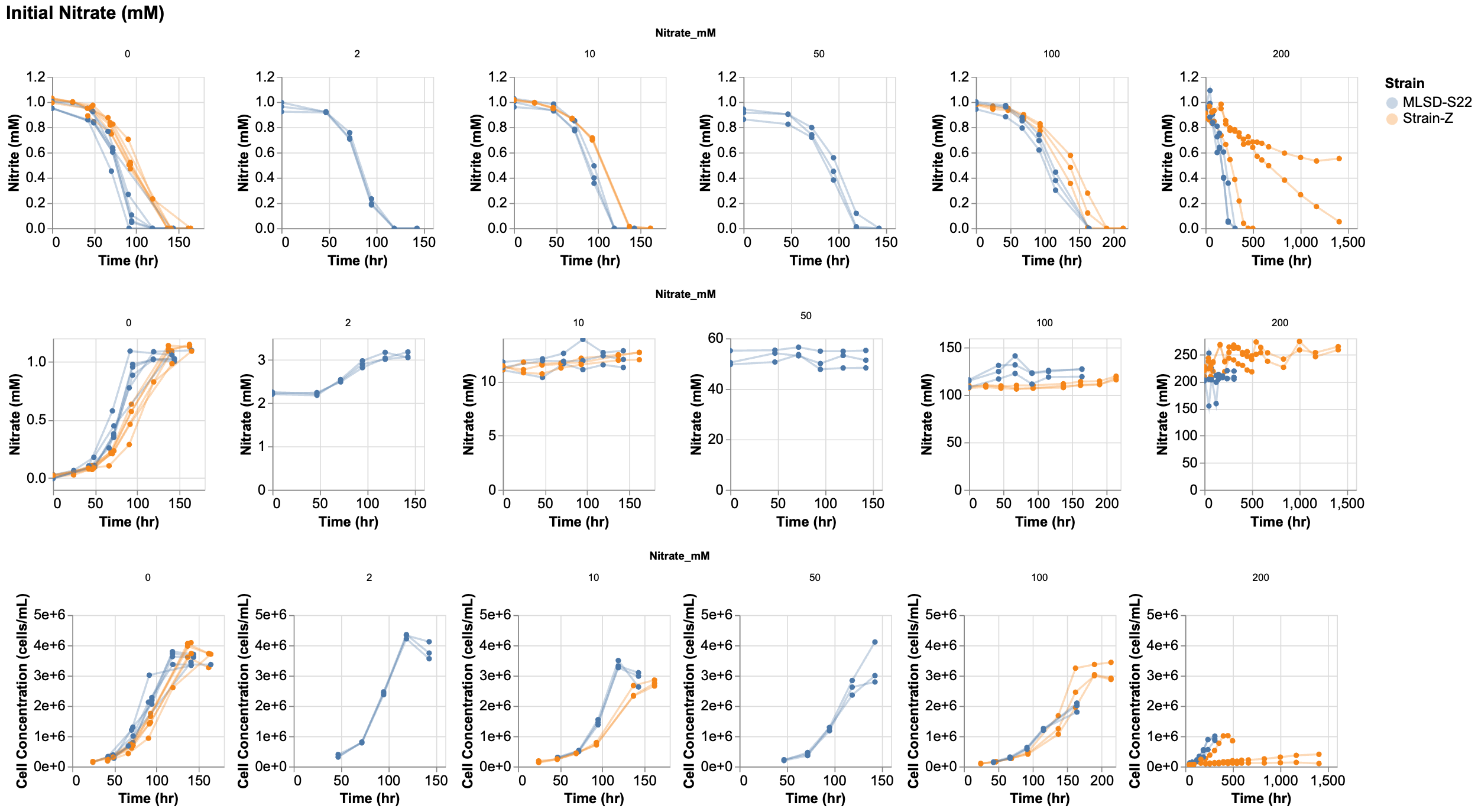


**Figure S2 – Growth curves for NO_3_^-^ inhibition experiments.** NO_2_^-^ consumption (top row), NO_3_^-^ accumulation (middle row), and cell counts (bottom row). Each column represents a different initial concentration of NO_3_^-^.


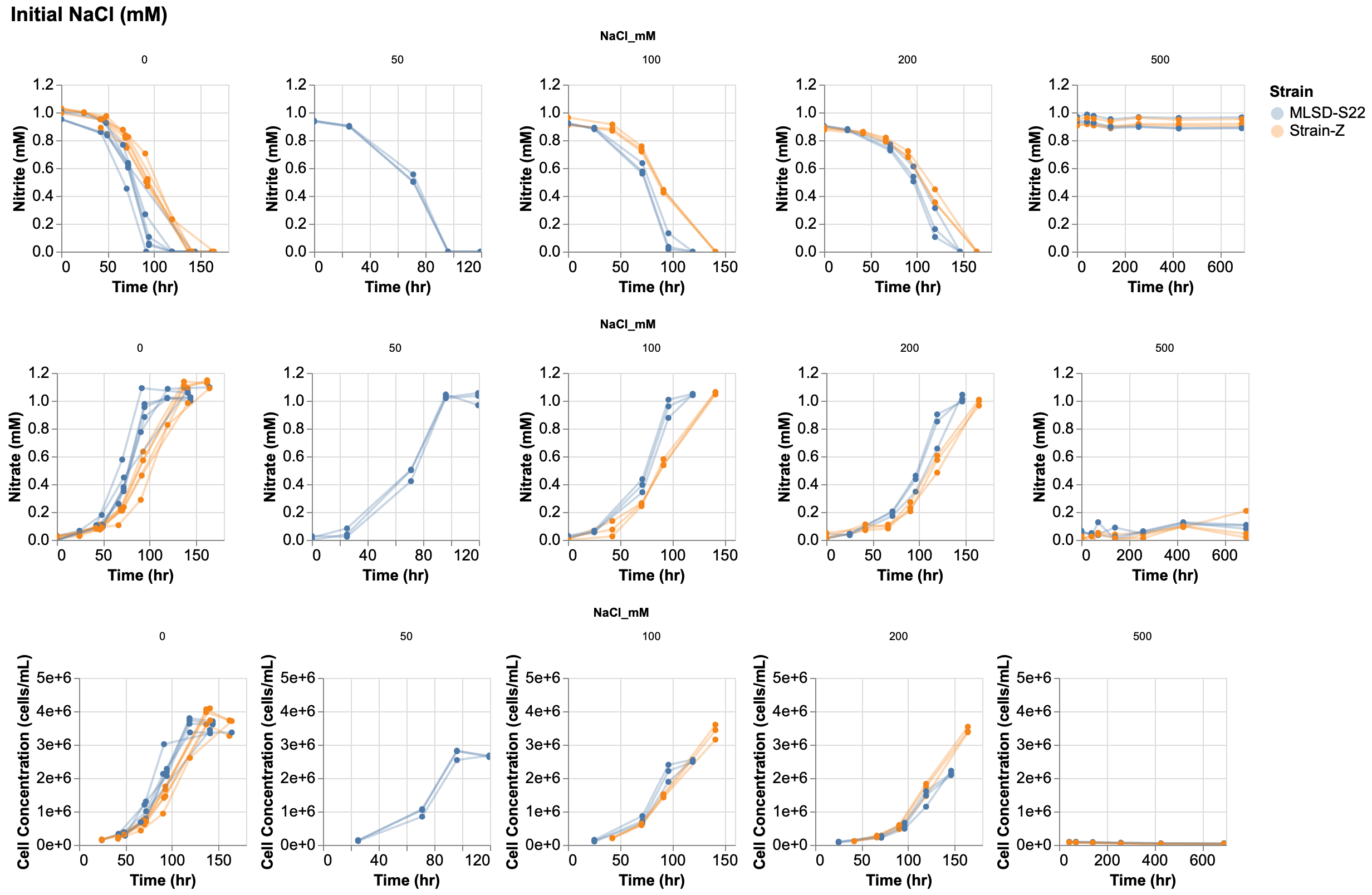


**Figure S3 – Growth curves for NaCl inhibition experiments.** NO_2_^-^ consumption (top row), NO_3_^-^ accumulation (middle row), and cell counts (bottom row). Each column represents a different initial concentration of NaCl.

**
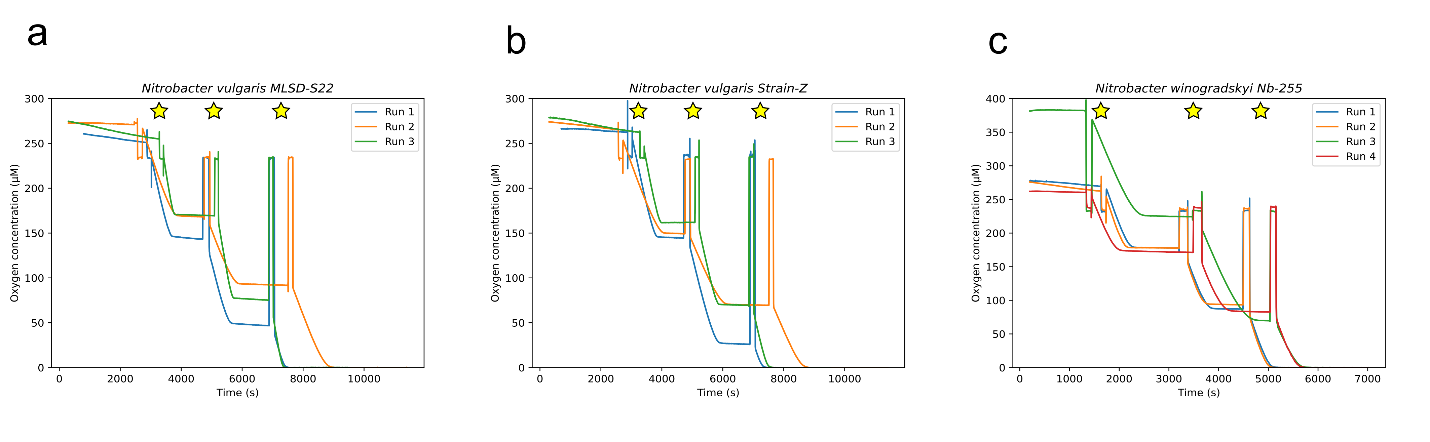
**

**Figure S4 – Raw O_2_ consumption traces from microrespirometry.** Traces from experiments with *N. vulgaris* strain MLSD-S22 (**a**), *N. vulgaris* strain Z (**b**), and *N. winogradskyi* strain Nb-255 (**c**). Stars show approximate times when NaNO_2_ was injected into the MR chambers. Large jumps in O_2_ concentration around these times correspond to the probe being removed from the chamber while the injection was taking place. The higher initial O_2_ concentration observed in *N. winogradskyi* (**c**) Run 3 is due to the injection of O_2_ after Run 1 which allowed for residual NO_2_^-^ to be oxidized and for the biomass to be further tested with additional injections. This approach was only used for *N. winogradskyi*.


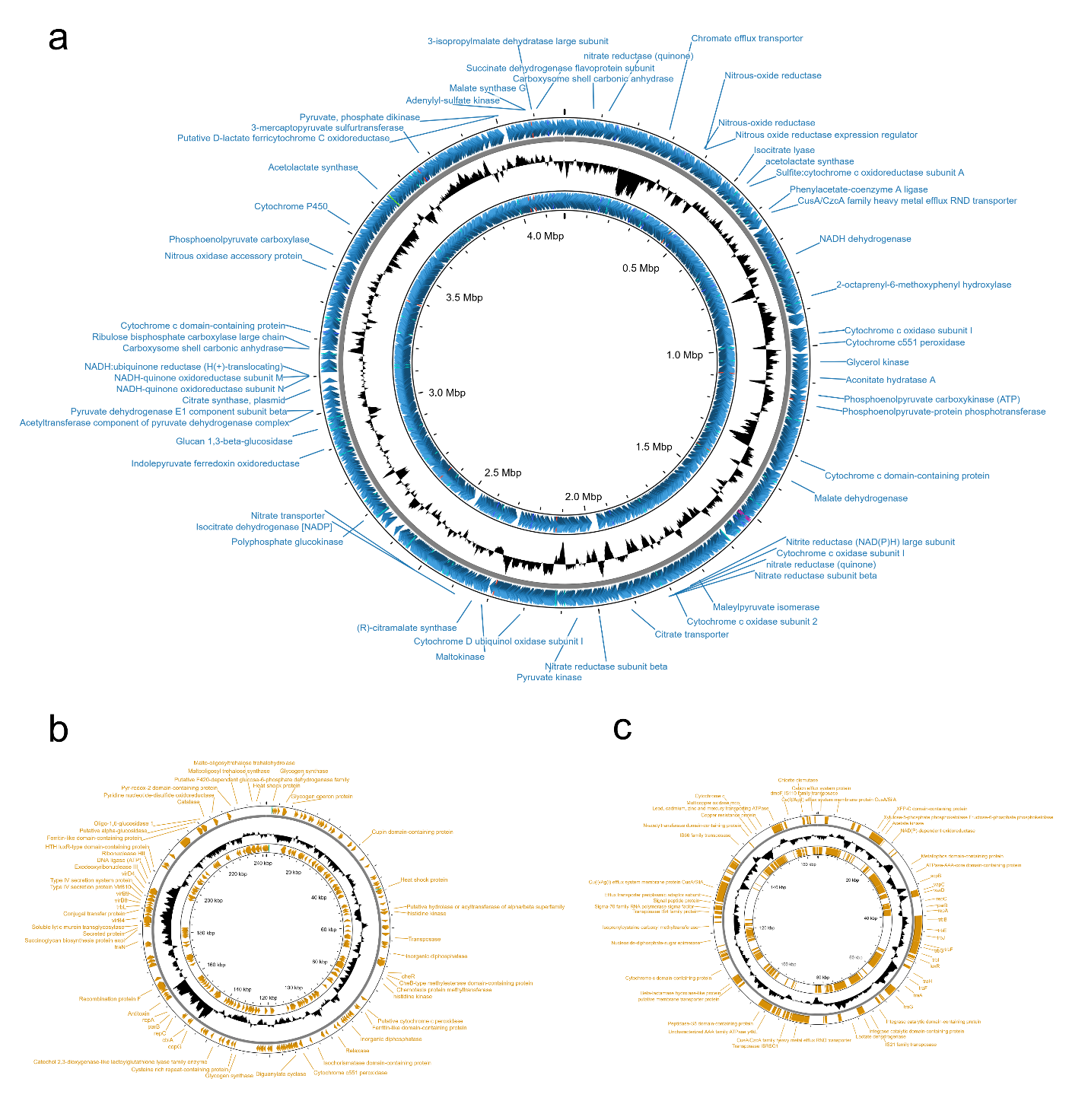


**Figure S5 – Strain MLSD-S22 genome and plasmids.** The main chromosome (**a**), the 242 kbp plasmid (**b**), and 164 kbp plasmid (**c**) with annotated genes of interest. Black plot indicates GC content deviation.


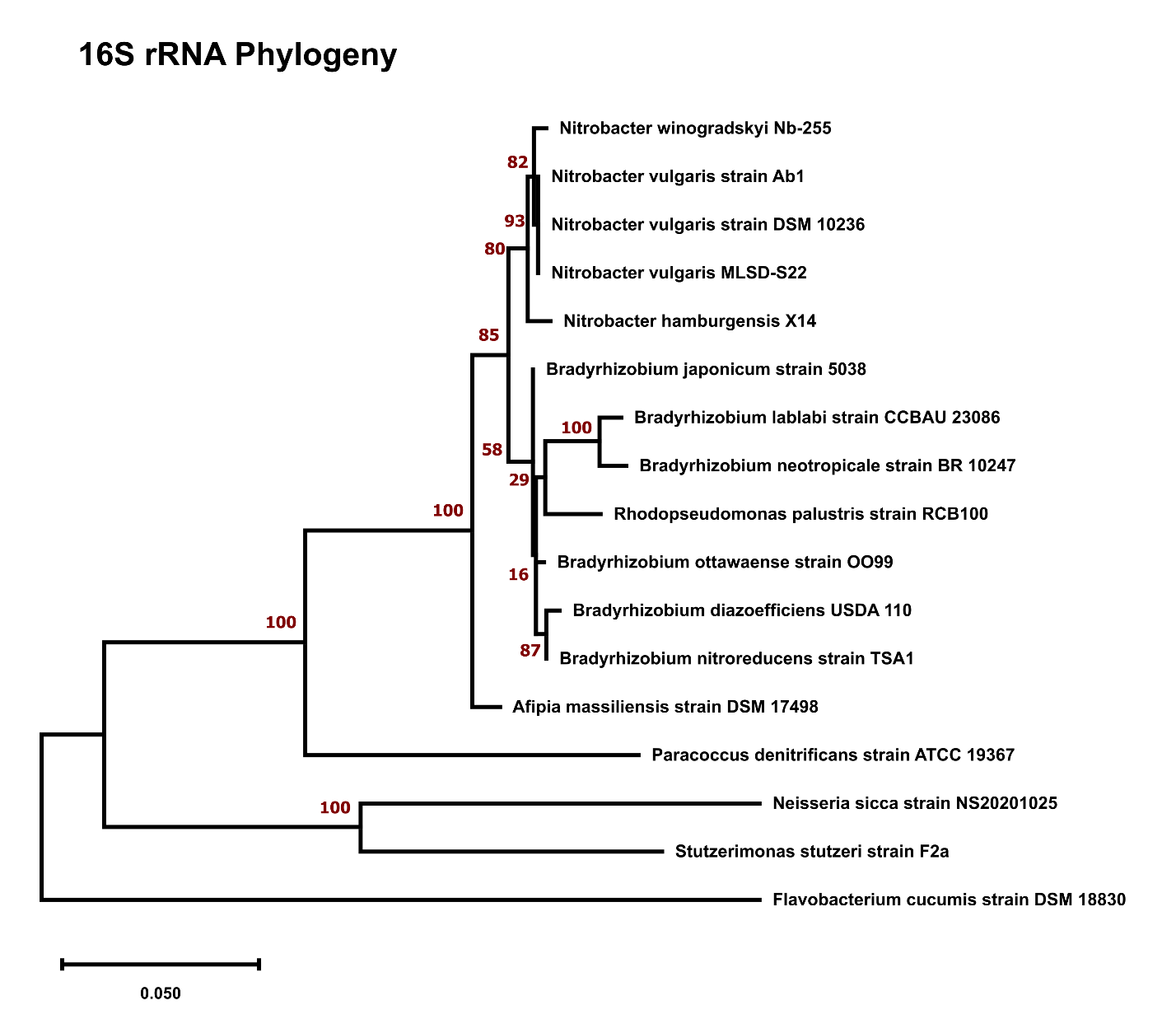


**Figure S6 – 16S rRNA Phylogeny for Nitrobacter and related organisms.** Maximum-likelihood tree of 16S rRNA genes. Red numbers indicate branch support from 100 bootstraps.


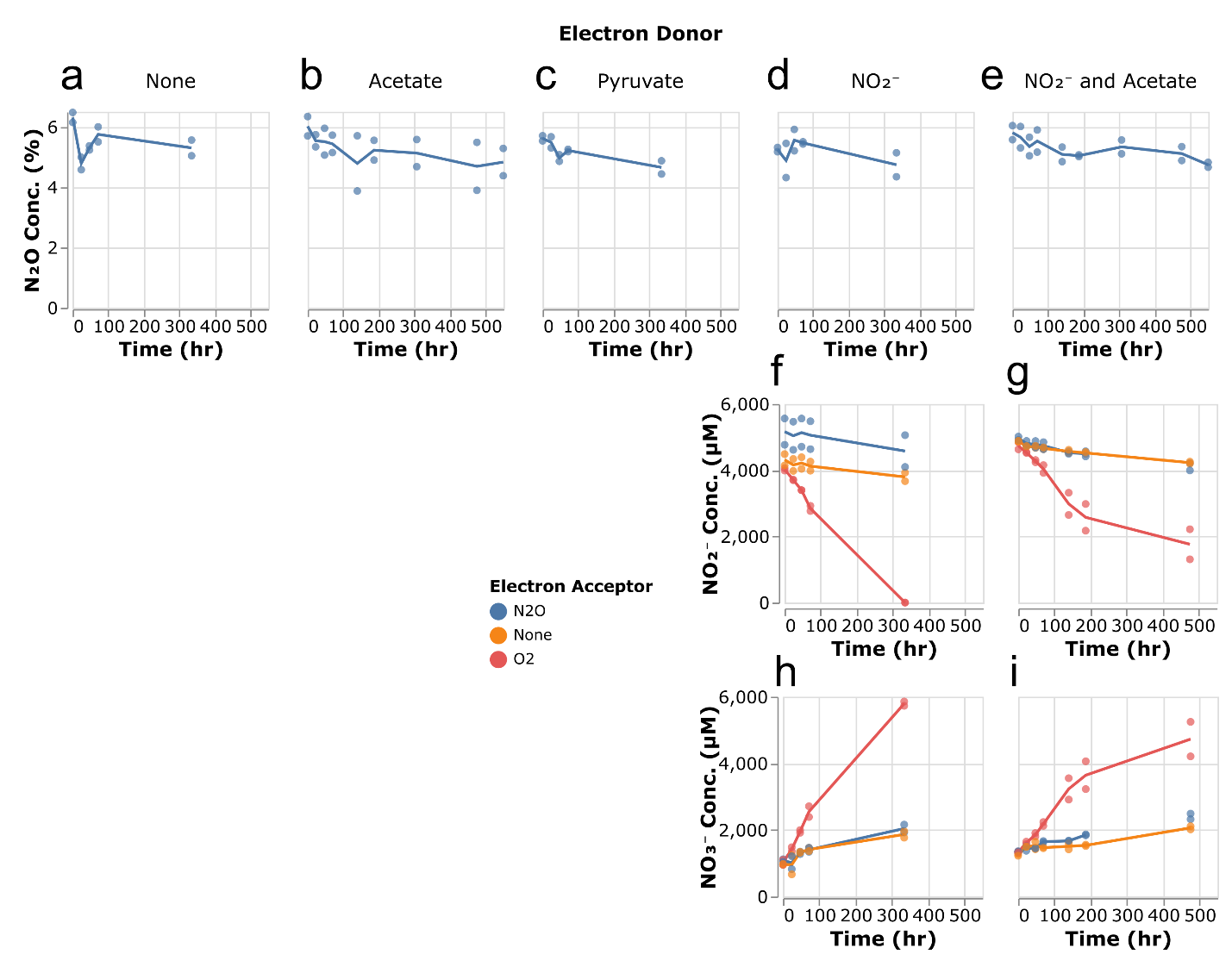


**Figure S7 – Testing N_2_O reduction potential.** Aerobically grown *N. vulgaris* strain MLSD-S22 was transferred to gas-tight tubes, flushed with N_2_, and N_2_O was injected. N_2_O concentration was monitored over time for incubations with various electron donors: None (**a**), 0.5 mM acetate (**b**), 0.5 mM pyruvate (**c**), 5 mM NO_2_^-^ (**d**), and 5 mM NO_2_^-^ in combination with 0.5 mM acetate (**e**). NO_2_^-^ and NO_3_^-^ concentrations were tracked for tubes with additional NO_2_^-^ added as an electron donor (**f**, **h**) and for tubes with NO_2_^-^ and acetate added together (**g**, **i**). For these cases, additional tubes were prepared with no additional electron acceptor added (shown in orange) and with O_2_ added (shown in red).

**References**

1. Kobayashi Y, Ninomiya T, Shiraishi Y, Kaneko A, Kuroiwa M, Suwa Y, Fujitani H. 2025. Physiological and genomic characterization of oligotrophic *Nitrobacter* isolated from a forest soil in Japan. Microbes Environ 40:1–9.

2. Lee U-J, Gwak J-H, Abiola C, Lee S, Yu J-S, Si O-J, Cho HJ, Quan Z-X, Kitzinger K, Daims H, Wagner M, Jung M-Y, Rhee S-K. 2025. Kinetic plasticity of nitrite-oxidizing bacteria containing cytoplasmic nitrite oxidoreductase https://doi.org/10.1101/2025.07.31.663499.

3. Both GJ, Gerards S, Laanbroek HJ. 1992. Kinetics of nitrite oxidation in two *Nitrobacter* species grown in nitrite-limited chemostats. Arch Microbiol 157:436–441.

4. Nowka B, Daims H, Spieck E. 2015. Comparison of oxidation kinetics of nitrite-oxidizing bacteria: Nitrite availability as a key factor in niche differentiation. Appl Environ Microbiol 81:745–753.

5. Hunik JH, Meijer HJG, Tramper J. 1993. Kinetics of *Nitrobacter agilis* at extreme substrate, product and salt concentrations. Appl Microbiol Biotechnol 40:442–448.

6. Laanbroek HJ, Bodelier PLE, Gerards S. 1994. Oxygen consumption kinetics of *Nitrosomonas europaea* and *Nitrobacter hamburgensis* grown in mixed continuous cultures at different oxygen concentrations. Arch Microbiol 161:156–162.

7. Su Z, Liu T, Guo J, Zheng M. 2025. Kinetic and physiological characterization of acidophilic *Nitrobacter* spp. in a nitrite-oxidizing culture. Environ Sci Technol https://doi.org/10.1021/acs.est.4c10020.

8. Blackburne R, Vadivelu VM, Yuan Z, Keller J. 2007. Kinetic characterisation of an enriched *Nitrospira* culture with comparison to *Nitrobacter*. Water Res 41:3033–3042.
